# Aortic root surgery with the CARDIOROOT vascular graft: results of a prospective multicenter post-market surveillance study

**DOI:** 10.1186/s13019-019-0914-y

**Published:** 2019-05-21

**Authors:** Giordano Tasca, Jaroslav Lindner, Laurent Barandon, Petr Santavy, Carlo Antona, Jan Burkert, Amando Gamba

**Affiliations:** 1 Operative Unit of Cardiac Surgery, Cardiovascular Department, ASST-Lecco, Ospedale “A. Manzoni”, Via Dell’Eremo 9/11, 23900 Lecco, Italy; 2 Chirurgická klinika kardiovaskulární chirurgie, General University Hospital, Prague, Czech Republic; 3 Department of Cardiac and Vascular Surgery, Centre Hospitalier Universitaire (CHU) de Bordeaux, Bordeaux, France; 4 Kardiochirurgická klinika, University Hospital Olomouc, Olomouc, Czech Republic; 5 Università degli Studi di Milano, Division of Cardio-Vascular Surgery, Department of Cardio-Cerebro-Vascular, ASST Fatebenefratelli-Sacco, Milan, Italy; 6 Dept. Cardiac Surgery, Klinika kardiovaskulární chirurgie, University Hospital Motol, Motol, Czech Republic

**Keywords:** Aortic root surgery, Vascular graft, Sinus of Valsalva, CARDIOROOT

## Abstract

**Background:**

Sparing Aortic Valve procedure requires to reproduction of the geometry of the physiologic anatomy of the aortic root. Thus, the materials adopted may make a difference. CARDIOROOT is a one-piece collagen-coated woven vascular graft with pseudo-sinuses, which was designed for use in the treatment of aortic root disease. We report the results of a prospective, multicenter, observational post-market surveillance study evaluating the safety and performance of the CARDIOROOT in patients requiring aortic root surgery.

**Methods:**

Patients with aortic root disease suitable for treatment with a vascular graft with pseudo-sinuses CARDIOROOT graft were eligible for participation. The enrolled patients were assessed intraoperatively, post-operatively, at discharge and at 1-year. Sites assessed complications at each visit, and recorded any reported adverse events. The study endpoint was mortality and complications through 1-year post-procedure.

**Results:**

Fifty-two patients were enrolled from 6 European centers. All procedures were technically successful. Operative mortality was 1.9%: one patient suffered hemorrhagic shock unrelated to the graft 1 day following surgery. At 1-year follow-up the survival rate was 96.2%, with a late death due to pneumonia 5 months post-procedure. Eleven serious adverse events occurred in 7 patients, which included cardiac complications (pericardial effusion, myocardial infarction and ventricular arrhythmia), infection (pericardial infection, deep sternal infection and superficial sternal infection), vascular disorders, including hemorrhagic shock and pleural effusion requiring drainage. Nine of the 11 events were deemed procedure-related by the local investigator, and all were deemed unrelated to the device. There were no reports of graft-related adverse events, infection, occlusion or graft failure.

**Conclusions:**

The results of this 1-year follow-up study showed that the CARDIOROOT vascular graft is safe and effective for the treatment of aneurysmal aortic root in immediate and mid-term follow-up. However, longer term follow-up is needed before conclusions can be made on the long-term safety and effectiveness.

**Trial registration:**

ClinicalTrials.gov Identifier: NCT01609270. Registered 31 May 2012.

## Background

Aortic root surgery is one of the most technically demanding procedures in cardiac surgery with higher risk of reoperation for bleeding and hospital mortality compared with a standard isolated aortic valve repair or replacement. When the aortic root disease is treated by a valve sparing technique, achieving a precise relationship among all the components of the aortic root and to restore near normal anatomy and function is imperative, and the materials used contribute greatly. The sparing aortic valve procedures have shown to be apt for the treatment of the aortic root diseases with excellent long-term results [1]. Although valve sparing procedures have been documented using completely straight tubular grafts, the use of a pre-formed graft with the shape of sinus of Valsalva has shown to allow for improved reproduction of normal aortic root physiology, and subsequently, physiologic results [2, 3]. Materials used to construct the prosthetic graft are important in order to optimize the graft’s performance once implanted. For example, construction using the woven technique compared to a knitting technique results in fabric less porous and less prone to dilate over time [4–6]. The woven technique consists of interlacing two sets of yarn (warp and weft) oriented at 90 degrees to one another. This confers peculiar mechanical properties and features to the graft. Among these features, there are a surface smoothness, easy handling, a low water permeability and suture retention strength [7]. The orthogonal arrangement of the yarn components yields a low radial compliance property. This arrangement results in a graft less prone to dilate immediately after the operation, as well as over time, compared with those made by means of knitted technique [4, 6].

In addition, all prosthetic grafts require a sealant to stop and minimize bleeding. The presence of a sealant comprised of collagen has shown superior healing properties with respect to fibroblasts, and a thinner inner healing tissue (intimal layer formation) compared with other sealants [8].

**Fig. 1:**
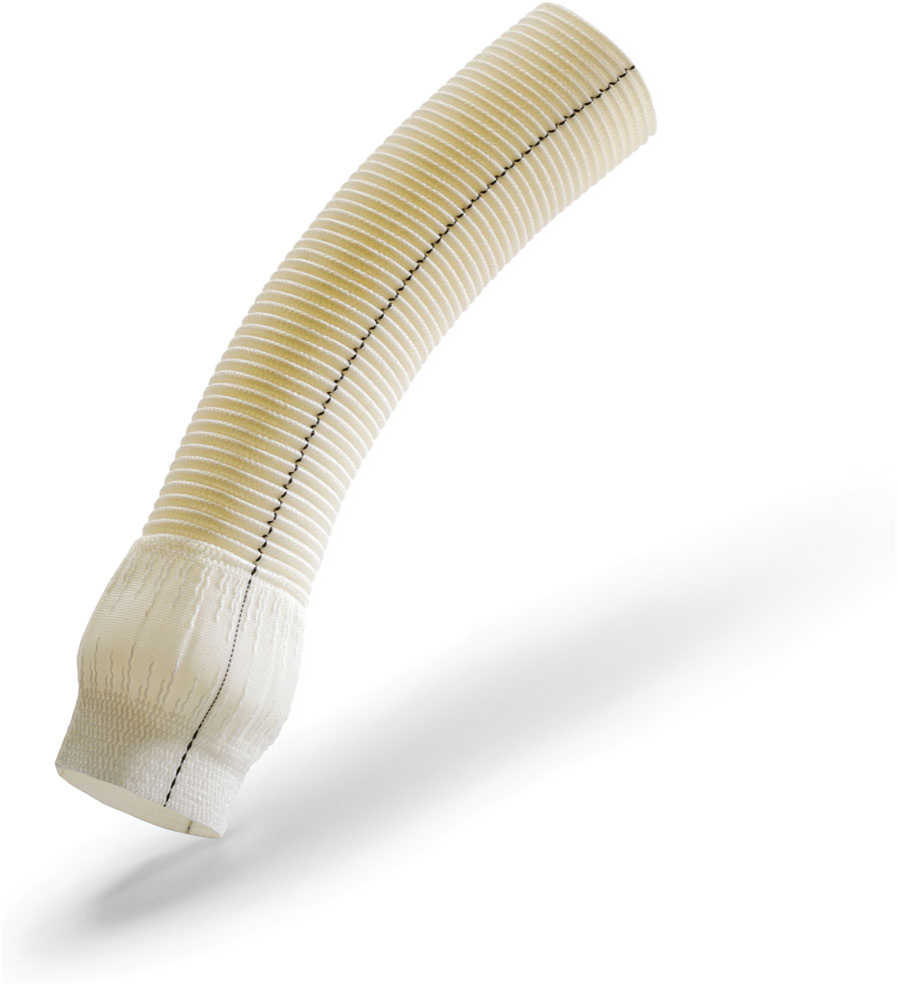
CARDIOROOT Device

CARDIOROOT (Intervascular SAS, La Ciotat, France) is one piece-design collagen-coated graft adopted in the repair or reconstruction of the aortic root. The unique design of the CARDIOROOT graft mimics the anatomy of the aortic root, and potentially allows physiologic flow and leaflets dynamics. This graft is made of woven polyester fabric coated with highly purified form of cross-linked Type I bovine collagen. The graft is constructed in three distinct regions including a Valsalva bulge, uncrimped with a drop shape, a woven tubular body above the Valsalva section and an uncrimped miniskirt collar underneath the Valsalva bulge. (See Fig. 1).

As a condition of commercial approval (Conformité Européene mark) for the CARDIOROOT vascular grafts, Intervascular SAS conducted a post-marketing surveillance registry of the CARDIOROOT vascular grafts in “real world” usage. This was a prospective, open-label, single-arm, multicenter, observational registry planned to enroll at least 50 subjects (Clinicaltrials.gov Reference Number: NCT01609270). The aim of the current study was to report the safety and performance of the CARDIOROOT vascular graft.

## Methods

### Patients

From November 2013 to June 2016, 52 patients (41 males, 79%, mean age 58, range 21 to 79 years) were treated at 6 study centers in Europe (France, Italy and Czech Republic). Patients with aortic root disease who were suitable, according to the local Investigators, to be treated by a CARDIOROOT vascular graft implantation were invited to participate in the registry. Patients were excluded based upon the contraindications as specified in the CARDIOROOT Instructions for Use, as well as patients that required consecutive aortic surgery, patients with extensive coronary artery disease, and patients who required urgent/emergent surgery. The local Institutional Ethics Committee approved the study protocol and patients provided written informed consent before surgery and inclusion in the registry.

Table 1 shows the main clinical characteristics of the population. Pre-operative echocardiographic assessment showed an average left ventricular ejection fraction of 60% ± 10%, aortic valve stenosis presence in 19% of patients, an aortic valve regurgitation (at least moderate) present in 19% and a bicuspid aortic valve in 10% of patients. All patients had an aortic root aneurysm and no patients had documented aortic dissection or aortic trauma.

**Table 1:** Demographics and pre-operative patient characteristics

Variable	Mean ± standard deviation (SD) or n/m (%)
Demographic variables	
Age (years), mean ± SD	58 ± 14
BMI (kg/m^2^)	26.6 ± 3.4
Gender (male), n/m (%)	41/52 (79)
Pre-operative clinical data	
Dyslipidemia, n/m (%)	15/52 (29)
Hypertension, n/m (%)	41/52 (79)
Diabetes, n/m (%)	0/52 (0)
Creatinine (mg/dl), mean ± SD	0.9 ± 0.2
Previous cardiac surgery, n/m (%)	4/52 (8)
New York Health Association, NYHA, functional status >II, n/m (%)	3/52 (6)
Heart failure, n/m (%)	8/52 (15)
Pre-operative cardiac characteristics	
Ejection fraction (%), mean ± SD	60 ± 10
Bicuspid aortic valve, n/m (%)	5/52 (10)
Valve stenosis, n/m (%)	10/52 (19)
Valve regurgitation ≥ II, n/m (%)	10/52 (19)

### Surgical procedure

Surgery was performed per clinician discretion. Operative approach chosen for all patients was a full conventional sternotomy. Average aortic cross clamp time was 93 ± 34 min and cardioplegia was delivered in anterograde fashion in 69% of patients, in retrograde in 27% or both in 4%. Valve surgery was performed (either an aortic valve replacement or leaflet repair) in 35 patients (67%), while other surgical procedures, other than coronary artery bypass grafting (6%), were performed in 15% of patients. Each patient received one CARDIOROOT vascular graft in the thoracic position. The 3 main CARDIOROOT sizes chosen by investigators were 28, 32 and 34 mm, which cover the majority of the sizes used (see Table 2).

**Table 2:** Operative results

Operative variable	Mean ± SD or n/m (%)
Cardiopulmonary bypass (min), mean ± SD	120 ± 41
Cross clamp time (min), mean ± SD	93 ± 34
Cardioplegia delivery, n/m (%)	
Anterograde	36/52 (69)
Retrograde	14/52 (27)
Both	2/52 (4)
Cardioroot diameter (body diameter)	
24 mm, n/m (%)	1/52 (2)
26 mm, n/m (%)	3/52 (6)
28 mm, n/m (%)	15/52 (29)
30 mm, n/m (%)	4/52 (8)
32 mm, n/m (%)	14/52 (27)
34 mm, n/m (%)	15/52 (29)
Valve surgery, n/m (%)	35/52 (67)
Coronary artery bypass grafting, n/m (%)	3/52 (6)
Other cardiac procedure, n/m (%)	8/52 (5)

### Evaluation and endpoints

Following surgical implantation of the CARDIOROOT graft, the enrolled patients were assessed intraoperatively, post-operatively, at discharge, during readmission (if applicable), at the 30-day follow-up visit, and at 1 year for survival status and any complications. Transthoracic echocardiography data was collected if performed per standard of care.

The study endpoint was the overall rate of deaths (all causes) and complications assessed at each visit up to 1-year post CARDIOROOT implant procedure. Participating sites completed a complications form for each assessment time point and also recorded any reported adverse events. Centralized monitoring was performed; source-document verification was limited.

### Statistical analysis

No formal sample size calculation was performed; registry sample size was based upon commitment with the Notified Body at the time of CE mark. Data are presented with number and percentage for all discrete variables and as average ± standard deviation for all continuous variables. Survival analysis was summarized descriptively by visit, and Kaplan-Meier estimates were provided. Two-sided confidence intervals (CIs) for the survival rate were calculated and presented using Greenwood’s formula. The number and percent of observed events (death or complication) at 30 days and 1 year were summarized based on subjects with available data along with the 95% exact binomial CIs for the percentages. Analysis was done using SAS version 9.4 (SAS Institute, Inc., Cary, NC, USA).

## Results

Fifty of the 52 patients completed the registry (i.e., completed 1-year visit after graft implantation). All procedures were technically successful; no procedures were abandoned or required re-operation. During the 30 postoperative days, 1 patient died due to hemorrhagic shock, yielding an operative survival of 98.1%. Another patient died at 5 months after the operation due to pneumonia, bringing the 1-year postoperative survival rate to 96.2%.

A total of 54 complications occurred in 32 patients throughout the follow-up period; 96% of these occurred within 30 days of the CARDIOROOT implant surgery. The most common was atrial fibrillation, reported in 17 patients (33%). Four patients required re-operation due to bleeding/tamponade (2 acute; 2 late). One patient required a re-operation for cardiac reasons, with sternotomy issues. Two patients suffered from sternal site infections (one superficial; one deep), and required packing/irrigation and wound vacuum-assisted closure. One patient experienced transient paralysis. Two patients reported pneumonia; one within 30 days and one at 5 months post-procedure. One patient experienced an unrelated gastro-intestinal event. Nineteen other complications were recorded but no details were provided. No specific adverse events relating to aortic regurgitation was reported. Transesophageal echocardiography was performed post-operative and at 1-year as per standard of care. Post-operatively, 38.5% of subjects had no aortic insufficiency, 48.1% had trace/trivial aortic insufficiency and 11.5% had mild aortic insufficiency. At 1 year, only 21/52 subjects (40.4%) had a recorded transthoracic echocardiography. Six [6] of these subjects had no aortic insufficiency; 9 had trace/trivial aortic insufficiency. Six [6] subjects had mild aortic insufficiency and no patients had moderate to severe aortic insufficiency.

Eleven serious adverse events were reported in 7 patients. The causes were assessed by the local Investigators and were deemed unrelated to the CARDIOROOT device. Nine of the 11 serious adverse events were deemed procedure-related. See Table 3.

**Table 3:** Serious Adverse Events

Serious Adverse Event	Incidence
Cardiac disorder	
Cardiac tamponade*	1 (1.9%)
Myocardial infarction*	1 (1.9%)
Pericardial effusion*	1 (1.9%)
Pericardial effusion/ Sternal dehiscence*	1 (1.9%)
Ventricular extrasystoles*	1 (1.9%)
Injury, poisoning and procedural complications	
Impaired healing/ Sternal wound healing delay*	1 (1.9%)
Postoperative thoracic procedure complication*	1 (1.9%)
Infections and infestations	
Pneumonia	1 (1.9%)
Pericardial infection*	1 (1.9%)
Musculoskeletal and connective tissue disorders	
Synovial cyst	1 (1.9%)
Vascular disorders	
Hemorrhagic shock (death)*	1 (1.9%)

* Deemed by the investigators to be related to the procedure

**Table 4:** Ethics Committees

Investigator / Site	Ethics Committee
01 Laurent Barandon, MD / Centre Hospitalier Universitaire (CHU) de Bordeaux Department of Cardiac and Vascular Surgery Bordeaux. France	Comité de Protection Des Personnes Sud-Ouest et Outre Mer III 146 Rue Léo Saignat, 33,000 Bordeaux, France
02 Prof. Jaroslav Lindner / Chirurgická klinika kardiovaskulární chirurgie General University Hospital in Prague Prague. Czech Republic	Etická komise Všeobecné fakultní nomocnice v Praze (Ethics Committee of the General University Hospital in Prague) Na Bojišti 1 128 08 Prague 2 Prague, Czech Republic Chairman: MUDr. Josef Šedivý, Csc. Date: 18 Oct 2012
03 Petr Šantavý, MD, PhD / Kardiochirurgická klinika University Hospital Olomouc Olomouc. Czech Republic	Fakultní Nemocnice Olomouc Etická komise Fakultní nemocnice Olomouc a Lékařske fakulty UP v Olomouci (Ethics Committee of the University Hospital Olomouc) I.P. Pavlova 6775 20 Olomouc, Czech Republic Chairman: doc. MUDr. Vladko Horčička, Csc. Approval date: 18 February 2013
04 Jan Burkert, MD, PhD / Klinika kardiovaskulární chirurgie University Hospital Motol, Dept. Cardiac Surgery Motol. Czech Republic	Etická komis FN MOTOL V úvalu 84 150 06 Praha 5 – Motol, Czech Republic Chairman: MU Dr. Jiří Škopek, PhD. Approval date: 13 November 2013
05 Amando Gamba MD / Giordano Tasca / Cardiac Surgery Ospedale Manzoni di Lecco Lecco, Italy	Comitato Etico Interaziendale delle Province di Lecco, Como e Sondrio Via dell’Eremo 9/1123900 Lecco, Italy Chairman: Dr. Antonio Giuseppe Cusumano Approval date: 12 Mar 2013
06 Prof. Carlo Antona / Ospedale Luigi Sacco Polo Universitario Dip Cardiocerebrovascolare Milan, Italy	Eitco Interaziendale Milano Area Ospedale Luigi Sacco Polo Universitario Milan. Italy Chairman: Prof. Emilio Trabucchi Approval date: 21 Nov 2014

## Discussion

The results of this 1-year follow-up study, which involved 52 patients, demonstrate that the CARDIOROOT vascular graft is effective in the treatment of the aortic root disease. The early and mid-term results have confirmed the safety with no anticipated or unanticipated events related to the CARDIOROOT device. Longer term follow-up is required to determine long-term effectiveness of the CARDIOROOT graft.

This registry also confirms the efficacy, as well as the versatility of the graft, given its adoption for both the aortic valve sparing and the Bentall procedure as composite graft.

**Fig. 2:**
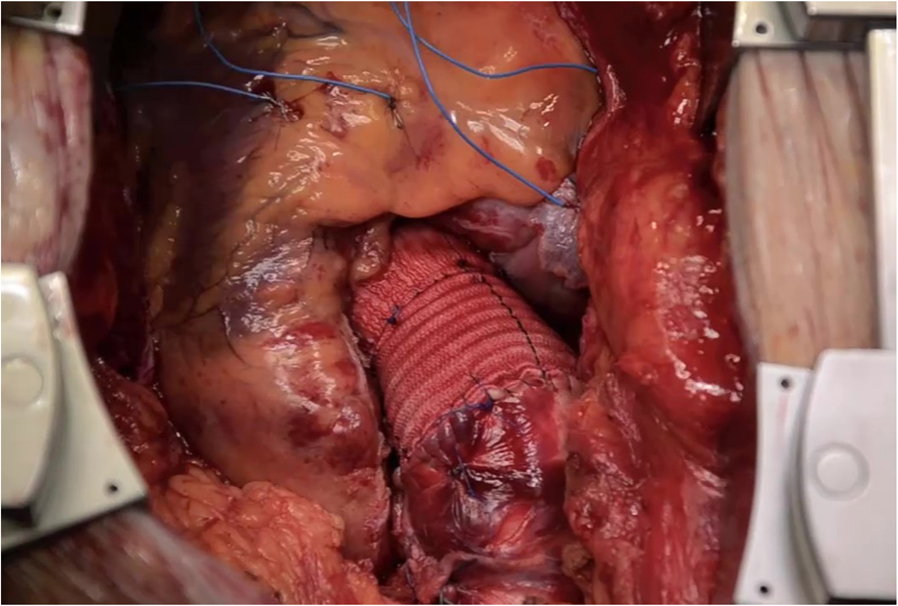
Cardioroot graft adopted in a Florida-Sleeve sparing aortic valve procedure

The unique “drop” shape of the CARDIOROOT graft mimics the physiologic anatomy sinuses of Valsalva. The bulge does not stretch when uploaded, making the Valsalva size more predictable. The shape may permit a more anatomical reconstruction of the sinuses with two potential benefits. In the case of the Florida-Sleeve procedure, where the entire aortic root is spared and wrapped by the graft [9] (Fig. 2), a more physiologic leaflet-sinus unit morphology restoration is expected, reproducing a near normal leaflet-sinus unit morphology [10]. That may ease the transferred part of the leaflet stress to the adjacent sinus of Valsalva wall [11] with a possible benefit on the durability of the procedure. While in the case of a composite graft, the presence of such sinus of Valsalva might have a positive impact on coronary flow dynamics [12], as well as on coronary stress when compared with the straight graft [13]. The three distinct regions of the CARDIOROOT facilitate graft handling and make the size and length of the root more predictable once uploaded by the blood pressure at the end of the operation. Maintaining a stable shape and the size of the aortic root is required to preserve the correct size ratio among the different components of the aortic root and in particular between the annulus and sinotubular junction. While this may have a favorable impact on valve or prosthesis function and should prolong the durability of a sparing aortic valve procedure, in the case of the composite graft, the stability in aortic root geometry may be useful in maintaining an optimized valve prosthesis fluid dynamics performance [14, 15].

While this registry was a multi-center, prospective registry, there are several limitations to note. The registry employed centralized monitoring and did not include full source document verification. Specific surgical information was not considered during data collection. The registry follow-up is limited to 1 year; further data for longer term follow-up is needed. The registry was also restricted by the nature of the procedures included by sites to aneurysmal disease and did not address dissection or other uses of the CARDIOROOT graft.

## Conclusions

The results of this registry demonstrated early and mid-term safety and effectiveness of the CARDIOROOT vascular grafts for the treatment of aneurysmal aortic root. The graft is fit for both the aortic sparing valve procedure and aortic root replacement as a composite graft.

## Abbreviations

CE: Conformité Européene
CI: Confidence interval
SD: Standard deviation
